# Clinical Consequences of Delayed Gastric Emptying With GLP-1 Receptor Agonists and Tirzepatide

**DOI:** 10.1210/clinem/dgae719

**Published:** 2024-10-17

**Authors:** Ryan J Jalleh, Mark P Plummer, Chinmay S Marathe, Mahesh M Umapathysivam, Daniel R Quast, Christopher K Rayner, Karen L Jones, Tongzhi Wu, Michael Horowitz, Michael A Nauck

**Affiliations:** Endocrine and Metabolic Unit, Royal Adelaide Hospital, Adelaide, SA 5000, Australia; Adelaide Medical School, The University of Adelaide, Adelaide, SA 5000, Australia; Adelaide Medical School, The University of Adelaide, Adelaide, SA 5000, Australia; Intensive Care Unit, Royal Adelaide Hospital, Adelaide, SA 5000, Australia; Endocrine and Metabolic Unit, Royal Adelaide Hospital, Adelaide, SA 5000, Australia; Adelaide Medical School, The University of Adelaide, Adelaide, SA 5000, Australia; Endocrine and Metabolic Unit, Royal Adelaide Hospital, Adelaide, SA 5000, Australia; Adelaide Medical School, The University of Adelaide, Adelaide, SA 5000, Australia; Southern Adelaide Diabetes and Endocrine Service, Flinders Medical Centre, Bedford Park, SA 5042, Australia; Diabetes, Endocrinology, Metabolism Section, Medical Department I, Katholisches Klinikum Bochum gGmbH, Sankt Josef-Hospital, Ruhr-University, D-44791 Bochum, Germany; Adelaide Medical School, The University of Adelaide, Adelaide, SA 5000, Australia; Department of Gastroenterology and Hepatology, Royal Adelaide Hospital, Adelaide, SA 5000, Australia; Endocrine and Metabolic Unit, Royal Adelaide Hospital, Adelaide, SA 5000, Australia; Adelaide Medical School, The University of Adelaide, Adelaide, SA 5000, Australia; Adelaide Medical School, The University of Adelaide, Adelaide, SA 5000, Australia; Endocrine and Metabolic Unit, Royal Adelaide Hospital, Adelaide, SA 5000, Australia; Adelaide Medical School, The University of Adelaide, Adelaide, SA 5000, Australia; Diabetes, Endocrinology, Metabolism Section, Medical Department I, Katholisches Klinikum Bochum gGmbH, Sankt Josef-Hospital, Ruhr-University, D-44791 Bochum, Germany; Institute for Clinical Chemistry and Laboratory Medicine, University Medicine Greifswald, D-17475 Greifswald, Germany

**Keywords:** GLP-1 receptor agonists, GLP-1/GIP dual receptor agonists, incretin mimetics, gastric emptying, tachyphylaxis, retained gastric content, aspiration

## Abstract

**Context:**

Glucagon-like peptide-1 (GLP-1) receptor agonists (RAs) are established therapeutics for type 2 diabetes and obesity. Among other mechanisms, they slow gastric emptying and motility of the small intestine. This helps to limit postprandial glycemic excursions and reduce chylomicron formation and triglyceride absorption. Conversely, motility effects may have detrimental consequences, eg, retained gastric contents at endoscopy or general anesthesia, potentially complicated by pulmonary aspiration or bowel obstruction.

**Data Acquisition:**

We searched the PubMed database for studies involving GLP-1RA therapy and adverse gastrointestinal/biliary events.

**Data Synthesis:**

Retained gastric contents at the time of upper gastrointestinal endoscopy are found more frequently with GLP-1 RAs but rarely are associated with pulmonary aspiration. Well-justified recommendations for the periprocedural management of GLP-1RAs (eg, whether to withhold these medications and for how long) are compromised by limited evidence. Important aspects to be considered are (1) their long half-lives, (2) the capacity of GLP-1 receptor agonism to slow gastric emptying even at physiological GLP-1 concentrations, (c) tachyphylaxis observed with prolonged treatment, and (d) the limited effect on gastric emptying in individuals with slow gastric emptying before initiating treatment. Little information is available on the influence of diabetes mellitus itself (ie, in the absence of GLP-1 RA treatment) on retained gastric contents and pulmonary aspiration.

**Conclusion:**

Prolonged fasting periods regarding solid meal components, point-of-care ultrasound examination for retained gastric content, and the use of prokinetic medications like erythromycin may prove helpful and represent an important area needing further study to increase patient safety for those treated with GLP-1 RAs.

Glucagon-like peptide-1 (GLP-1) receptor agonists (RAs) and the dual incretin receptor agonist tirzepatide have a major, and increasing, impact on the current management of type 2 diabetes (T2D) and obesity. GLP-1 RAs not only have a potent glucose-lowering effects but also promote weight loss in obese individuals, reduce the risk of cardiovascular and renal disease, and may improve metabolic dysfunction-associated steatotic liver disease. GLP-1RAs impact gastric, intestinal, and gallbladder motility, which can be beneficial (eg, through slowing glucose and lipid entry into the circulation after meals) but may also increase the risk of adverse consequences (eg, retained gastric contents, potentially resulting in pulmonary aspiration during endoscopy or induction of general anesthesia and gallbladder and biliary complications). In this review, we summarize the state-of-the-art knowledge regarding the beneficial and detrimental consequences of the effects of GLP-1RAs on gastrointestinal motility with the intent of formulating sound clinical recommendations.

## Literature Search Strategy

We searched the PubMed database for publications with the following search terms in the title or abstract: “glucagon-like peptide-1,” “GLP-1,” “exenatide,” “lixisenatide,” “liraglutide,” “dulaglutide,” “albiglutide,” “semaglutide,” “efpeglenatide,” and “tirzepatide”; each of these terms in combination with “gastric emptying,” “gastroparesis,” “chylomicron,” “motility,” “retained,” “gastric content,” “pulmonary aspiration,” “small bowel obstruction.” In addition, retrieved manuscripts were searched for additional relevant references.

## Beneficial Consequences of GLP-1RA Effects on Gastric Emptying

### Reduction in Postmeal Glycemic Excursions

Elevation of postprandial glycemia (PPG) is characteristically the initial defect in glucose intolerance (1) and is a challenging aspect of the management of T2D (2). Monnier at al reported that PPG is a dominant contributor to overall glycemia in T2D (as assessed by measurement of hemoglobin A_1c_), especially in well-controlled diabetes [calculated to contribute approximately 70% when hemoglobin A_1c_ is ≤7.3% (3)]. A few robust clinical trials have explored the impact of reducing PPG on vascular complications, with evidence of beneficial impacts on both microvascular (eg, retinopathy) and macrovascular (eg, cardiovascular) disease (4, 5).

Gastric emptying is now recognized as a major determinant of postprandial glycemic excursions in both healthy subjects (6) and people with diabetes (7) and accounts for about a third of the variance in the initial rise in glucose postoral challenge (ie, first 15-30 minutes) (6). In people with normal glucose tolerance, the early (30- or 60-minute) rise in plasma glucose following a 75 g oral glucose challenge is directly proportional to the rate of emptying, while this relationship is reversed at 120 minutes (the time point conventionally used in the diagnosis of diabetes). However, in people with impaired glucose tolerance or T2D, a “right-ward shift” is observed, such that a direct positive relationship is observed even beyond 60 minutes (8). There is increasing interest in the 60-minute postload glucose concentration as a predictor of future T2D and a treatment target (9), and it is intuitively likely that the rate of gastric emptying is a key determinant. Modulating gastric emptying, such as accelerating emptying using a prokinetic agent such as erythromycin or retarding it with morphine (10) or nutrient preloads (eg, protein) (11), has been shown to acutely increase or decrease PPG excursions, respectively, in T2D.

### Native GLP-1 and Gastric Emptying

Acute IV administration of GLP- 1 slows gastric emptying profoundly and dose-dependently (12), accompanied by relaxation of the gastric fundus, increased gastric compliance, inhibition of antral contractility, and increased pyloric tone (13, 14). This dose-dependent slowing of gastric emptying represents a major mechanism contributing to PPG lowering by exogenous GLP-1 and, in healthy humans, “outweighs” its insulinotropic effects such that postprandial insulin secretory responses are suppressed rather than stimulated (15, 16). Not surprisingly, a specific GLP-1 receptor antagonist, exendin [9–39] amide, accelerates gastric emptying in individuals without diabetes with proportional rises in plasma glucose and insulin (17), indicating that physiological postprandial concentrations of GLP-1 are sufficient to slow gastric emptying substantially.

The observation that IV GLP-1 leads to a reduction in plasma glucose after a meal in individuals with T2D, while a rise in plasma glucose was observed after subsequent meals (18), suggested there may be tachyphylaxis for the effects of GLP-1 to slow gastric emptying. Nauck et al evaluated the effect of exogenous IV GLP-1 (7-36 amide), delivered at 0.8 pmol.kg^−1.^l^−1^ for 8 hours, on gastric emptying (by a dye dilution technique) of 2 successive meals consumed at baseline and after 4 hours of GLP-1 exposure (19). Both acute and 4-hour exposure to GLP-1 slowed gastric emptying markedly, but the degree of deceleration was less for the second meal. Subsequently, Umapathysivam et al compared the effects of acute, intermittent (2 4.5-hour infusions separated by 20 hours) and continuous (20 hours) IV GLP-1 (0.8 pmol.kg^−1.^min^−1^) with placebo on gastric emptying (with scintigraphy, the gold-standard measurement) in a double-blind crossover study in healthy humans (20). Both acute and intermittent exposure were shown to slow gastric emptying potently and comparably. Although still substantial, the slowing of emptying was diminished with continuous exposure. Taken together, these observations suggest that a reduction in the deceleration of gastric emptying may occur even within 4 hours of administration of exogenous GLP-1RAs (20).

### Short- vs Long-acting GLP-1RAs and Evidence for Tachyphylaxis for Effects on Gastric Emptying

**Figure 1.**
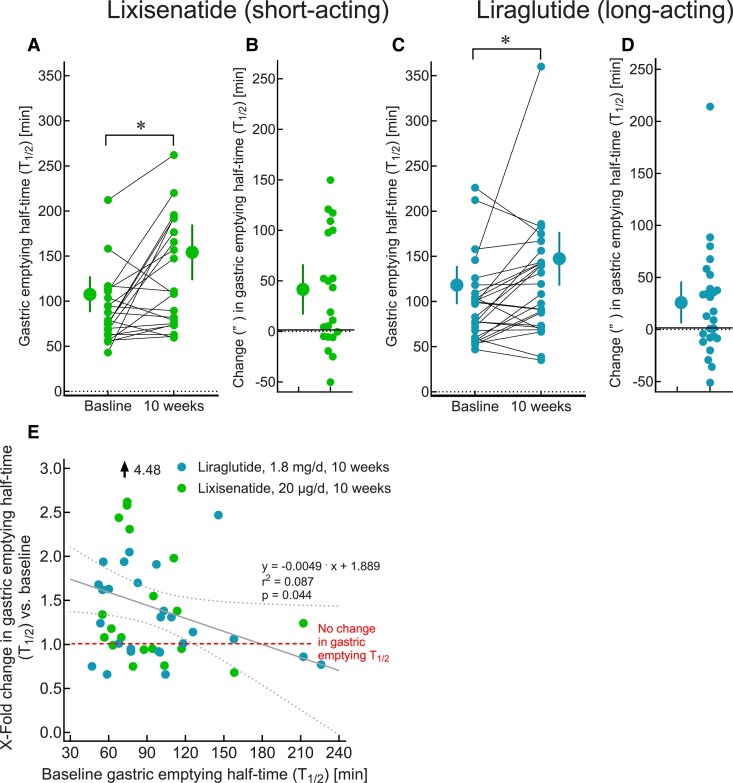
Effects of GLP-1RAs (lixisenatide and liraglutide) on gastric emptying in T2D. Gastric emptying (half-time) of a standardized meal (∼1175.7 kJ, egg, white bread, margarine, and water) in participants before and after treatment for 10 weeks with lixisenatide (20 µg per day; A) or liraglutide (1.8 mg per day; C). (B) and (D) show the change in gastric emptying half-times from baseline with lixisenatide (B) and liraglutide (D) treatment). (E) The x-fold change in the gastric emptying half-time (y-axis) is plotted vs the baseline gastric emptying half-time (solid components, measured by a 6-hour ^13^C-octanoate breath tests analyzed using Wagner-Nelson equations, x-axis), comparing the effects of the short-acting GLP-1RA lixisenatide (20 µg once daily over 10 weeks, n = 21) and the long-acting GLP-1RA liraglutide (1.8 mg once daily over 10 weeks, n = 26), with baseline gastric emptying (before GLP-1RA treatment). Linear regression analyses (regression equation, r^2^, and related *P*-value) indicate a significant reduction in GLP-1RA effect to slow gastric emptying in subjects with relatively slower gastric emptying at baseline for both lixisenatide and liraglutide. Data are from Quast et al (40). Statistical analysis: linear regression analysis (regression line, r^2^, and *P*-value). Please refer to Supplementary Table S1 for additional information on the effect of GLP-1RAs on gastric emptying in subjects with T2D and obesity. Abbreviations: GLP-1, glucagon-like peptide-1; RA, receptor agonist; T2D, type 2 diabetes.

**Figure 2.**
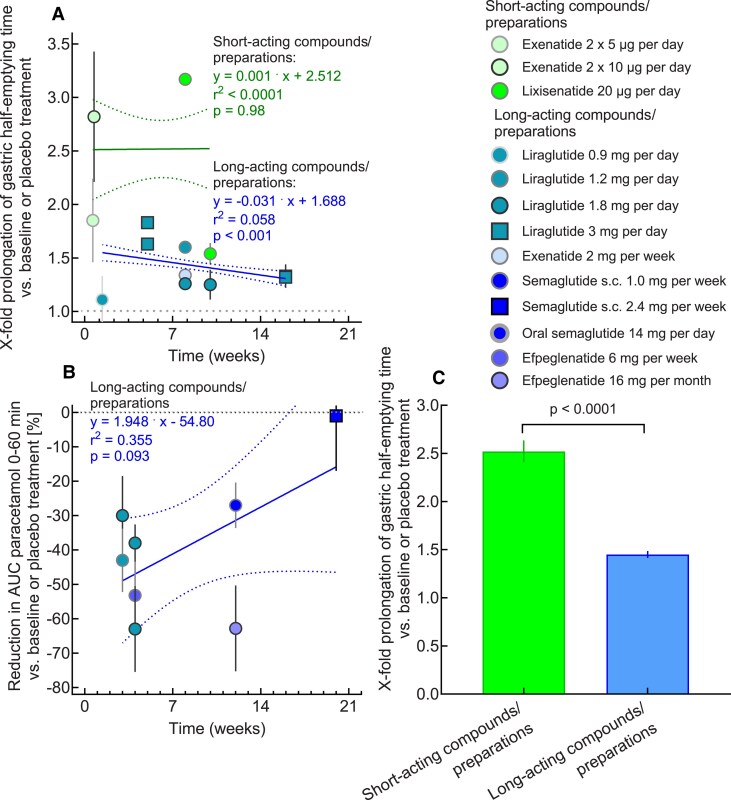
Duration of therapy with GLP-1RAs and their effects on gastric emptying in studies employing scintigraphy or ^13^C-octanoate breath tests (for the determination of gastric emptying half-times for solid meal components; A) or paracetamol absorption (expressed as the reduction in the area under the curve of paracetamol plasma concentrations during the first hour after a meal test; B), both plotted against the time after initiating GLP-1RA treatment. Pooled effect sizes for data displayed in panel (A) are shown separately for short-acting (exenatide twice a day, lixisenatide) and long-acting GLP-1RAs (C). Linear regression analysis (regression equations, r^2^, and related *P*-values) revealed a significant reduction over time in the prolongation of gastric emptying half-times for long-acting GLP-1RAs supported by a similar trend for paracetamol absorption (A) and indicating tachyphylaxis but not for short-acting GLP-1RAs (B). The overall stronger effect on gastric emptying shown for short-acting GLP-1RAs in panel (C) may in part be the result of a lack of tachyphylaxis for these compounds. However, given the limitations of the paracetamol absorption test, interpretation must be considered in circumspect. Please refer to Supplementary Table S1 for more data on the effect of GLP-1 receptor agonists on gastric emptying in subjects with type 2 diabetes and obesity. Abbreviations: GLP-1, glucagon-like peptide-1; RA, receptor agonist.

GLP-1RAs have been divided into “short-acting” (eg, exenatide twice a day and lixisenatide) or “long-acting” (eg, liraglutide, dulaglutide, exenatide QW, semaglutide), based on whether their plasma concentrations return to near-zero levels between subcutaneous (SC) administrations or remain pharmacologically effective over at least 24 hours. Clinical studies reporting effects of short- and long-acting GLP-1RAs on gastric emptying in individuals with T2D or obesity are summarized in Figs. 1 and 2 and in Supplementary Table S1 (21). Figure 1 illustrates measurements of gastric emptying with the short-acting GLP-1RA lixisenatide and the long-acting GLP-1RA liraglutide (22). The considerable interindividual variation in baseline gastric half-emptying times is evident (Fig. 1A and 1C) as well as the variable slowing induced by lixisenatide (Fig. 1B) and liraglutide (Fig. 1D). Notably, neither lixisenatide nor liraglutide slowed gastric emptying in every individual.

Twice a day exenatide (5 vs 10 µg twice daily for 5 days) was shown to slow gastric emptying by scintigraphy in T2D in a dose-dependent manner, associated with a reduction in postmeal plasma glucose and peak insulin concentrations (23). The overall magnitude of both slowing of gastric emptying and glucose-lowering were marked but variable and dependent on the baseline rate of gastric emptying and less prominent in those with slow baseline gastric emptying (23). Furthermore, in a small scintigraphic study (n = 20), IV exenatide was shown to slow small intestinal transit with an associated attenuation in blood glucose rise in individuals with and without T2D (24). Similar analyses for lixisenatide and liraglutide confirm little further deceleration of gastric emptying in patients with abnormally delayed and even relatively slower gastric emptying at baseline (Fig. 2E). Lorenz et al reported slowing of gastric emptying, assessed by a stable isotope (^13^C octanoic acid) breath test, following 4 weeks of treatment with lixisenatide, accompanied by a reduction in postprandial glucose excursions (25).

Lixisenatide’s effects on gastric emptying of a liquid oral glucose load has been studied after a single SC dose of 10 µg per day (26) and after 8 weeks of treating with 20 µg per day (27), both in inidividuals with and without T2D. Gastric emptying was markedly delayed under both conditions, irrespective of a diagnosis of diabetes mellitus. Remarkably, there were considerable interindividual differences regarding the effects of lixisenatide on gastric emptying, with some subjects experiencing only minimal gastric emptying over the study period, such that half-emptying times could not be determined. Thus, lixisenatide, as a representative of short-acting GLP-1 RAs, profoundly decelerated gastric emptying of a liquid meal, with little difference over time (single dose vs 8 weeks), supporting the absence of substantial tachyphylaxis (26, 27).

After sustained exposure to long-acting GLP-1RAs, tachyphylaxis for the effects on gastric emptying has been observed (19). Nevertheless, residual slowing of gastric emptying remains, for example with liraglutide (1.8 mg per day) after 4 weeks (28) or exenatide once weekly (2 mg) after 8 weeks (27), (Figs. 1 and 2.) Clinical studies evaluating the effects of GLP-1RA treatment for periods of up to 21 weeks (the longest period reported so far) on gastric emptying are summarized in Supplementary Table S1 (21) and displayed as Fig. 2. Linear regression analysis did not illustrate a reduction over time in the effects of short-acting GLP-1RAs twice-a-day exenatide and lixisenatide on gastric emptying, while with long-acting GLP-1RAs (liraglutide, exenatide once a week, semaglutide SC and oral, and efpeglenatide), there appears to be a decline in the magnitude of this slowing over time. The fact that the effect sizes overall appear to be greater for short- vs long-acting GLP-1RAs may, in part, reflect the presence of tachyphylaxis with long- but not with short-acting formulations. To what degree the effects of long-acting GLP-1RAs on gastric emptying persist over durations longer than, eg, 16 weeks and how this affects postprandial glucose lowering cannot be answered based on the available data and needs to be studied.

### Interindividual Variation Regarding Tachyphylaxis of Gastric Emptying Effect of GLP-1 RAs

Effects of liraglutide (3 mg per day) on gastric emptying of a mixed meal in individuals with obesity has been studied at baseline and after 5 as well as 16 weeks. Mean gastric half-emptying times are prolonged after 5 weeks of treatment but significantly less so after 16 weeks (29). However, a secondary analysis of the same data shows substantial interindividual variability in the effects of liraglutide on gastric emptying and regarding the temporal pattern of effects of this long-acting GLP-1 RA (30).

### GLP-1RAs—Relationship Between Gastrointestinal Symptoms and Gastric Emptying (Central vs Peripheral Effect)

The quality of data relating to the effect of GLP-1RA on gastric emptying is highly variable, largely due to the continued use of imprecise measurement techniques (31) like the paracetamol (acetaminophen) absorption technique, which continues to be used extensively (32-34). While inexpensive and simple to perform, its limitations are partitioning in the liquid phase and the inability to assess gastric emptying of solids reliably (31, 35, 36). Scintigraphy remains the gold-standard technique but is limited by cost, availability, and exposure to ionizing radiation (37). The stable isotope breath test represents an acceptable alternative that is noninvasive, simple to perform, and readily available and has been validated against scintigraphy (38, 39, 41).

Similarly, the evaluation of gastrointestinal symptoms such as nausea, vomiting, and diarrhea, which occur frequently with GLP-1RAs (42) and have a major influence on the adherence to such treatment over time, have often been assessed in clinical trials solely via participant self-report, which is known to be unrelaible (eg, symptoms may not be volunteered due to embarrassment or poor recall) (43, 44). Validated instruments, developed to diagnose and differentiate functional gastrointestinal disorders, such as the Patient Assessment of Upper Gastrointestinal Disorder Symptom Severity Index questionnaire or the Gastroparesis Cardinal Symptom Index should be more widely used in clinical trials with GLP-1RAs (43, 44).

In longstanding type 1 diabetes (T1D) and T2D, there is a high prevalence of upper and lower gastrointestinal symptoms (37); gastric emptying is delayed in 30% to 50% of individuals, and intragastric meal distribution is also frequently abnormal (37). It was traditionally believed that gastrointestinal symptoms were a direct outcome of a delay in gastric emptying. The majority of studies that have evaluated the relationship between the effects of GLP-1RAs on gastric emptying and gastrointestinal symptoms in health, obesity, or T2D have found no or weak correlations (45-49). Gastric emptying may be markedly delayed in the absence of symptoms, and in some individuals with severe symptoms, gastric emptying may be normal or accelerated (11). Uncomplicated T1D and T2D, as well as obesity, are often associated with more rapid rather than delayed gastric emptying (11). In the absence of GLP-1 RA treatment, the presence of symptoms is not a direct indicator of delayed gastric emptying (27, 50). However, a recent study studying SC semaglutide obesity without T2D reported a significant relationship between gastrointestinal symptoms and gastric emptying after (51).

In summary, the presence or absence of gastrointestinal symptoms cannot be used to diagnose or exclude the presence of delayed gastric emptying. Instead, this condition must be specifically measured using suitable techniques such as scintigraphy or the stable isotope breath test.

### Effects of GLP-1RAs on ad Libitum Food Intake

**Figure 3.**
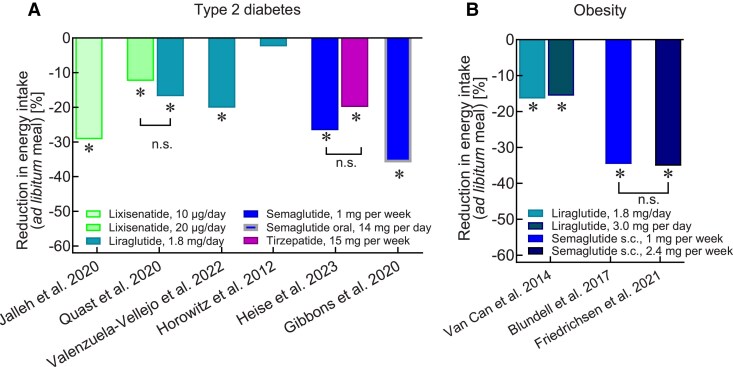
Effects of GLP-1RAs and the GIP/GLP-1 dual receptor agonist tirzepatide on ad libitum caloric intake of test meals in subjects with T2D (A) and obesity without T2D (B). The percent reduction vs baseline energy intake (± SEM) is displayed. Asterisks indicate a significant reduction (*P* < .05); n.s. indicates not significant (for GLP-1 receptor agonists studied head to head in some studies). Please refer to Supplementary Table S2 for additional information about the effect of GLP-1RAs on ad libitum energy intake and perceptions of hunger, satiation, fullness, and prospective food consumption in individuals with either T2D or obesity without T2D. Abbreviations: GLP-1, glucagon-like peptide-1; RA, receptor agonist; T2D, type 2 diabetes.

Based on the initial observation that low-dose intracisternal administration of GLP-1 reduces food intake acutely in rodents (52, 53), and similar findings of exogenous IV administration of GLP-1 in healthy humans (54), GLP-1RAs have been found to reduce body weight substantially in both T2D and obesity. The majority of studies indicate that both short- and long-acting GLP-1RAs reduce energy intake in health (27), obesity (55-59), T1D (60), and T2D (22, 27, 61-63) with only 1 study (with liraglutide in T2D) failing to show a significant reduction in energy intake compared to placebo (28) [Fig. 3; Supplementary Table S2 (21)]. Figure 3 summarizes dedicated clinical studies measuring the effect of various GLP-1RAs on ad libitum energy intake [Supplementary Table S2 (21)]. Since gastric distension (both of the proximal and distal stomach) can lead to reduced food/energy intake (64, 65), the question arises as to whether the slowing of gastric emptying, via increased gastric distension, contributes to the regulation of appetite and energy intake.

### Do Slower Gastric Emptying and Greater Gastric Distension With GLP-1RAs Contribute to a Reduction in Energy Intake?

In individuals without diabetes, sensations of bloating/fullness are more closely associated with emptying from the distal stomach and antral area as measured by ultrasound (50). There is limited information about the effect of GLP-1RAs on intragastric meal distribution. In individuals with and without T2D, the marked slowing of gastric emptying induced by an acute dose of lixisenatide (10 µg) is associated with increased retention in both the proximal and distal stomach (27). In this study, subsequent energy intake at a buffet meal was related to the retention in the distal stomach during placebo in healthy human subjects, an effect not observed in T2D or after lixisenatide (27). The lack of a relationship between energy intake and changes in gastric emptying and intragastric distribution in individuals treated with lixisenatide is consistent with the concept that effects on appetite are primarily centrally mediated (27). Similarly, no correlation between gastric emptying (measured using the isotope breath test) and energy intake was found in a separate trial (22). Accordingly, a possible mechanism of action of GLP-1 RAs to reduce appetite and energy intake may involve interaction with neurons in the hypothalamus either directly (66, 67) or via the nodose ganglion (68), rather than a secondary consequence of slowing gastric emptying.

### Effects on Chylomicron Formation and Postmeal Lipoprotein Profiles

GLP-1 signaling also modulates chylomicron formation, secretion, and clearance. Chylomicrons are large triglyceride-rich lipoproteins that contain a unique surface apolipoprotein B-48 (ApoB-48) (69) and are assembled in enterocytes from dietary lipids. Accordingly, slowing of gastric emptying and/or small intestinal transit induced by GLP-1 or GLP-1RAs can limit the delivery of dietary lipids for the synthesis of chylomicrons. Moreover, both GLP-1 (70) and GLP-1RAs [eg, exenatide (71), albiglutide (72), and liraglutide (73)] have been shown to inhibit gallbladder emptying and refilling and to potentially reduce the discharge of bile into the small intestine, interrupting the subsequent emulsification and digestion of dietary lipids. Of note, exenatide is effective in suppressing the production of chylomicrons and the rise of apoB-48 in the circulation, following administration of a lipid-containing “meal” directly into the small intestine in individuals without diabetes (74). This establishes that the effect is, at least in part, independent of gastric emptying. Conversely, in rats, antagonism of GLP-1 signaling by exendin [9-39] augments lymph flow and triglyceride and ApoB-48 concentrations in the lymphatic fluid after an intestinal lipid load (75). Moreover, in mice, genetic deletion of GLP-1 receptors enhances ApoB-48 secretion in the absence of changes in gastric emptying (76). These observations support the concept of a direct physiological effect of GLP-1 signaling to govern intestinal chylomicron formation. In line with these concepts, dipeptidyl peptidase-4 (DPP-4) inhibitors, which only modestly increase (about double) the concentrations of intact, biologically active GLP-1 and have no effects on gastrointestinal motility, are capable of reducing postprandial triglyceride and ApoB48 responses substantially (77, 78). Both lixisenatide (79) and liraglutide (80) have also been reported to increase chylomicron triacylglycerol clearance. All GLP-1RAs [reviewed in (81)] and, more recently, the dual incretin receptor agonist tirzepatide, have been shown to lower postprandial triglyceride and ApoB-48 levels significantly (82). These effects may well be relevant for the cardiovascular benefits associated with GLP-1RA treatment and, as discussed, appear to be both dependent and independent of the effects on gastric emptying.

## Potentially Detrimental Consequences of Slowed Gastric Emptying and Other Effects of GLP-1 RAs on Gastrointestinal Motility

### RAs and the Risk of Small Bowel Obstruction or Ileus

IV infusions of native GLP-1 that achieve either physiological or supraphysiological plasma concentrations are associated with inhibition of the migrating motor complex (83) and a decrease in the number and amplitude of duodenal pressure waves during both fasting and intraduodenal lipid infusion (14). Similar phenomena are evident with the use of exenatide (24), which has been shown to inhibit small intestinal motility and increase gastrointestinal transit time in individuals with and without T2D (84). While the mechanisms by which GLP-1 and GLP-1RAs affect intestinal motility remain to be defined, they appear to inhibit gastrointestinal motor function through both parasympathetic (vagal) afferents and direct central nervous stimulation (85-87).

These observations have stimulated concerns that treatment with GLP-1RAs or DPP-4 inhibitors may be associated with an increased risk of bowel obstruction or ileus. Although these adverse effects have not been evident in large randomized controlled studies involving these agents, there are several case reports linking these potentially serious conditions to the use of GLP-1RAs (88, 89) and the dual incretin receptor agonist tirzepatide (90) in people with and without T2D.

Several studies systematically investigating the potential association of GLP-1RAs with bowel obstruction utilized large medical databases. Accordingly, limitations in data quality, including reporting bias, should be considered in interpretating the outcomes. A study using 2 data repositories from the United Kingdom included data from 25 617 subjects treated with GLP-1RAs and compared the rate of bowel obstruction with 67 261 subjects treated with sodium-glucose cotransporter-2 (SGLT-2) inhibitors using propensity-score matching (91). This study reported a weighted incidence rate of 1.9 vs 1.1 per 1000 person-years (hazard ratio [HR] 1.69, 95% confidence interval [CI] 1.04-2.74) for GLP-1RAs. Surprisingly, the same study also found an increased risk for treatment with DPP-4 inhibitors, which raise intact GLP-1 concentrations only modestly. Furthermore, confidence intervals were wide for individual GLP-1RAs, and no events were reported with semaglutide, which otherwise is the most effective selective GLP-1RA. Interestingly, the risk appeared to increase with the duration of exposure to GLP-1RAs, with the greatest risk observed after 1.6 years (91).

A similar finding was reported in an analysis of adverse drug reactions using the World Health Organization pharmacovigilance database VigiBase (92). Of 501 244 adverse reactions reported with diabetes drugs between 2007 and 2018, only 698 (1.4%) involved “gastrointestinal stenosis and obstruction,” of which 216 (∼ 0.04% of the total) were treated with GLP-1RAs. Almost 30% of the events were reported by nonmedically trained individuals, which may affect the diagnostic accuracy of these findings. While an increased odds ratio (OR) was evident for bowel obstruction with GLP-1RAs (OR 3.05, 95% CI 2.54-3.66), reporting rates varied substantially from year to year (eg, odds ratios for GLP-1RAs varied between ∼ 1 in the years 2008, 2010, 2013, and 2014 vs ∼ 3 in 2009, 2011, 2012, and 2015). Considering the increased availability and use of GLP-1RAs in people with and without T2D in recent years, the lack of a corresponding rise in reports is worth mentioning.

Based on a Bayesian analysis of the US Food and Drug Administration averse event reporting system database from April 2005 to December 2021, a modest but statistically significant increase in ileus cases (information component, a measure of the strength of the correlation between GLP-1RA use and ileus [IC_025_], ∼ 0.12-0.77) was found for some GLP-1RAs, although semaglutide (IC_025_ −0.77) was not associated with an increase in events (93). In contrast, another study applying similar methodology in the same database for events between January 2018 and September 2022 reported a significant association of ileus cases with semaglutide (IC_025_ 3.04 [0.11 lower one-sided 95% CI]), but not for any other GLP-1RAs (94).

Another study investigated the risk of gastrointestinal adverse events (biliary disease, pancreatitis, bowel obstruction, or gastroparesis) in a random sample of 5411 subjects with obesity (identified by International Classification of Diseases code) using the PharMetrics Plus database (95). Individuals using the GLP-1RAs liraglutide (76.6% of the cohort) and semaglutide (11.3% of the cohort) were compared to new users of bupropion-naltrexone (12.1% of the cohort). While the cohort was relatively small and sample sizes differed substantially (87.9% GLP-1 vs 12.1% comparator), the authors reported an increased risk of bowel obstruction in those treated with GLP-1RAs (HR 4.22 [95% CI 1.02-17.40]). When the analysis was performed regardless of a history of obesity, the risk was lower (2.44 [95% CI 1.00-5.95]). Of note, the overall incidence of small bowel obstruction was low (1.4%), and no case was reported in the semaglutide group.

The most recent study investigated nationwide registers from Sweden, Denmark, and Norway, comparing 121 254 new users of GLP-1RAs with 185 027 new users of SGLT-2 inhibitors between 2013 and 2021 (96). Subjects were included at the date of filling their first prescription, and groups were matched using Cox regression with propensity score matching. In contrast to previous studies, the incidence of bowel obstruction (including ileus, intussusception, volvulus, neurogenic bowel, megacolon, and other types of bowel obstruction) was not found to differ between SGLT-2 inhibitors and GLP-1RAs but tended to be nonsignificantly protective against these diagnoses (adjusted HR 0.83 [95% CI 0.69 to 1.01]). Strengths of this study lie in the high validity of the source and the diagnoses.

It should be appreciated that, in general, the use of DPP-4 inhibitors in these studies was associated with a higher risk of intestinal obstruction than GLP-1RAs (91, 92, 96, 97). This is surprising, since the overall incidence of gastrointestinal adverse events is much higher with the latter class (98). This raises the question as to whether, if there is an effect, the underlying mechanisms do not relate to inhibitory effects on motility, since DPP-4 inhibitors appear to have little to no effect on this parameter in either obese subjects or those with T2D (99).

In summary, available data on the association of bowel obstruction or ileus with the use of GLP-1 RAs are inconsistent, and no definitive conclusions can be made. While some studies suggest up to 3-fold increase in risk, others report no association.

### Risk of Retained Gastric Contents in Patients Using GLP-1RAs or Tirzepatide Presenting for Endoscopy or General Anesthesia

**Figure 4.**
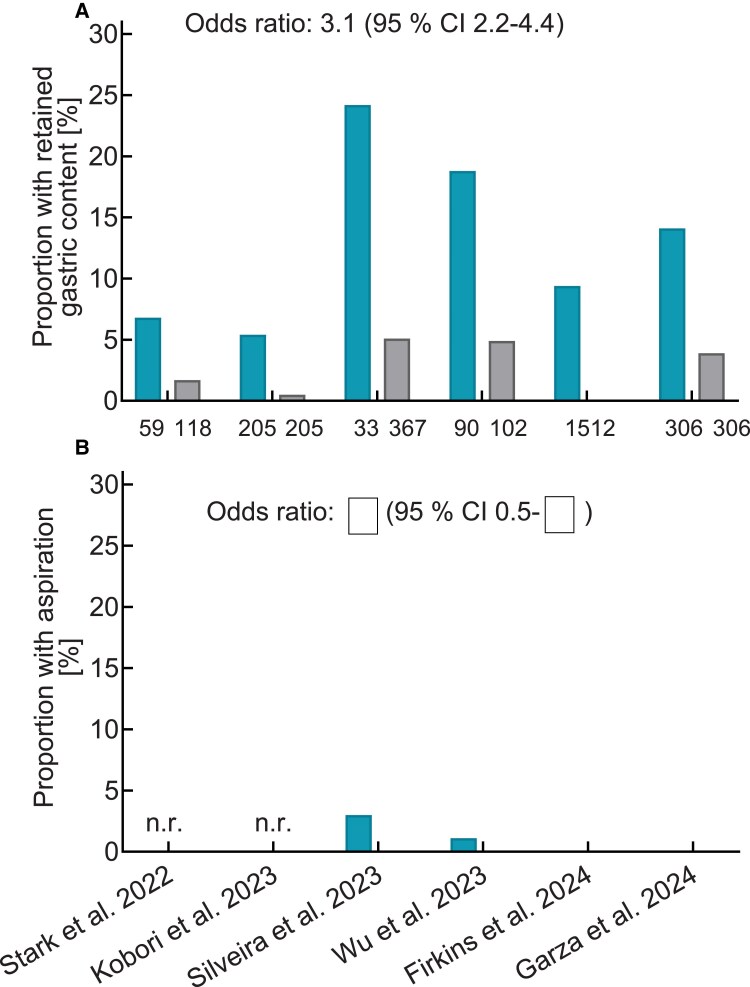
Outcomes of retrospective studies summarizing monocentric experience with the incidence of residual gastric content (A) and evidence for aspiration (B) in the context of upper gastrointestinal endoscopy in participants (T2D and obesity) either treated with various GLP-1RAs (blue bars) or not treated with GLP-1RAs (grey bars). More subjects treated with GLP-1RAs had diabetes. In studies reporting both residual gastric content and aspiration, it can be concluded that 0.2% of those with residual gastric content also had evidence for aspiration (data not shown). For more information, please see Supplementary Table S3. Abbreviations: GLP-1RA, glucagon-like peptide-1 receptor agonist; T2D, type 2 diabetes.

Retrospective studies suggest that individuals on GLP-1-based therapy (exenatide, liraglutide, dulaglutide, semaglutide, or tirzepatide) appear to be at an increased risk of having retained gastric contents [Supplementary Table S3 (21); Fig. 4]. In the largest study of 35 183 patients who underwent oesophago-gastro-duodenoscopy, among 922 (2.6%) patients using a GLP-1RA compared to nonusers, there was a 4-fold increase in the risk of retained gastric contents (13.6% vs 2.3%, *P* < .0001) and a 4-fold rate of aborted procedures (*P* < .0001) (100). The majority of patients using GLP-1RAs in this study had T2D (82%), but outcomes were consistent even after stratifying the analysis by diabetes status, suggesting that an increased risk for retained gastric contents was present irrespective of diabetes status. Multiple other single-center retrospective analyses have consistently showed that GLP-1RA use is associated with an increased risk of the presence of retained gastric contents on upper endoscopy (51, 101-104) [Supplementary Table S3 (21); Fig. 4]. Residual food may increase the risk of perioperative pulmonary aspiration (105). This is a rare complication of general anesthesia, with an incidence of approximately 1 per 3000 elective surgery cases, resulting in death in approximately 1 per 70 000 cases (106). The risk of pulmonary aspiration in emergency surgery is considerably higher at 1 per 900 cases, in part because these patients are usually not fasted.

### Retained Gastric Content When the Stomach Should be Empty: Relation to Dysfunctional Meal-related Gastric Emptying or Impaired Interdigestive Motility?

**Figure 5.**
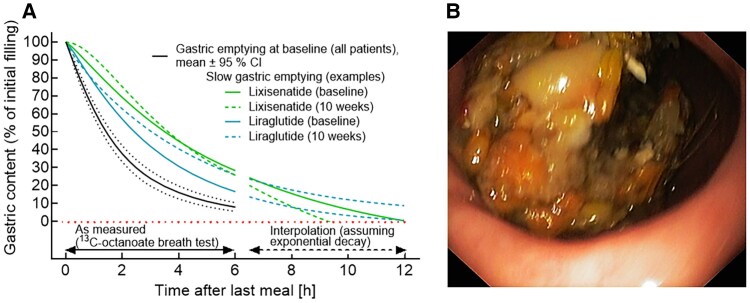
Indirect evidence to demonstrate that slow pot-prandial gastric emptying should not result in substantial gastric retention 12 hours after the last meal. (A) Six-hour gastric emptying tests (^13^C- octanoate breath for labeling solid meal components) analyzed using Wagner-Nelson equations were performed in 47 T2D subjects at baseline (black curves, with dotted lines indicating ± 95% confidence intervals). Results from subjects with substantially decelerated gastric emptying (at baseline or 10 weeks after initiating treatment with lixisenatide [20 µg/d] or liraglutide [1.8 mg/d]) are also shown. Gastric emptying curves between 6.5 and 12 hours after meal intake were interpolated assuming an exponential decay (from an initial plateau of 100%) based on the measurements during the initial 6 hours. Even those with slow gastric emptying achieved a gastric content near 0% by 12 hours, which would be a typical interval between a last meal (eg, dinner at 7 Pm) and an early procedure (endoscopy or general anesthesia) in the morning of the following day. Data used for display and modeling are from Quast et al (40). (B) Residual gastric content from food ingested on the previous day (or earlier) as visualized in the gastric antrum by upper gastrointestinal tract endoscopy (courtesy Diabetes Center Bad Lauterberg). Abbreviations: T2D, type 2 diabetes.

If retained gastric contents are observed in the morning (the typical time for endoscopic procedures and induction of anesthesia) after an >12-hour fast (after dinner), the question arises regarding whether this can be related to the typical gastric emptying curve following meal ingestion. Gastric emptying is usually measured over periods of up to 6 hours, when >90% of solid meal components should have emptied, as shown in Fig. 5. This 6-hour period coincides with the time during which labeled glucose enters the circulation after oral glucose or mixed meals (107). In addition to mean values, individual gastric emptying curves are shown for those subjects with T2D, who had the slowest gastric emptying at baseline or after treatment with lixisenatide or liraglutide (green; Fig. 5A). When their gastric emptying was predicted for postmeal hours 6 to 12, even those patients should have had minimal residual gastric content by 12 hours. Thus, one could doubt that deceleration of the typical course of postprandial gastric emptying is what explains retained gastric contents.

**Figure 6.**
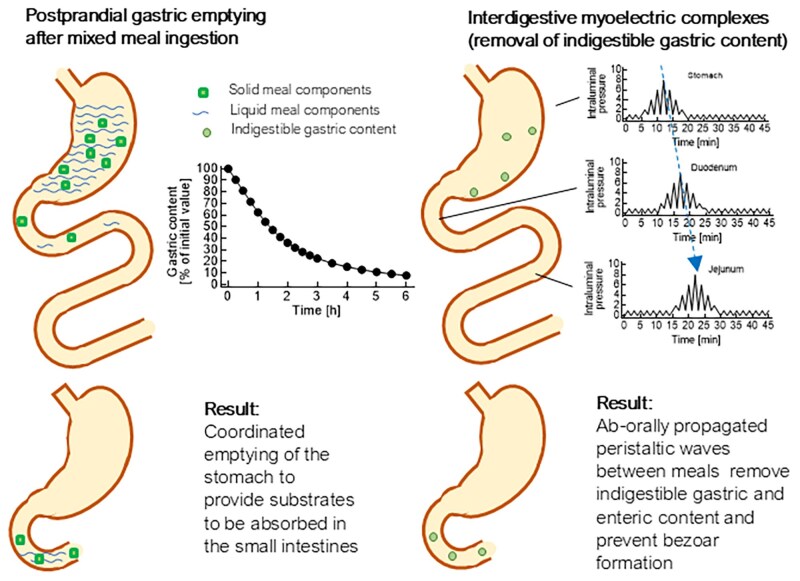
Schematic diagram illustrating postprandial gastric emptying and interdigestive motility (especially migrating motor complex phase 3) that clear the upper gastrointestinal tract of residue, including indigestible, gastric content. While meal-related gastric emptying normally extends over a period of approximately 6 hours, after which the bulk of a meal has emptied, interdigestive gastrointestinal motility occurs at regular intervals between meals and is interrupted by meal ingestion. GLP-1 and GLP-1RAs decelerate meal-related gastric emptying (see Figs. 1 and 2 and Supplementary Table S1). Exogenous GLP-1 at both physiological and pharmacological doses has been shown to reduce the frequency of migrating motor complex phase 3 in individuals without diabetes (14). Studies with GLP-1RAs have not been reported. Abbreviations: GLP-1, glucagon-like peptide-1; RA, receptor agonist.

Another mechanism that might better explain the finding of residual gastric content under these conditions (illustrated in Fig. 5B) could be an interference of GLP-1RA treatment with the interdigestive migrating motor complex (MMC), particularly phase 3 of this complex, which is composed of pressure waves often originating in the stomach and propagated segment by segment toward the duodenum and jejunum (108), which is thought to clear the remnants of the preceding meal including indigestible gastric content from the stomach (108, 109). The differences between postmeal gastric emptying and the “housekeeping” function of interdigestive MMC activity are illustrated in Fig. 6. The occurrence of the MMC, including phase 3 activity, is suppressed by exogenous GLP-1 (14), even at near-physiological intact GLP-1 concentrations. Influences of GLP-1RAs on this form of gastrointestinal motility have not been adequately studied or reported. Therefore, a role for reduced interdigestive motility induced by GLP-1RA treatment in the increased risk of retained gastric content remains a hypothesis to be further substantiated by dedicated studies.

However, this might explain why, despite having fasted for a median duration of 15.8 hours (intequartile range 12.6-18.5) from solid food and 11.0 hours (interquartile range 5.6-14. 6) from liquids (103), there was still a higher incidence of retained gastric contents in GLP-1RA users. Same-day colonoscopy has been observed to have a protective effect (prevalence ratio 0.25, 95% CI 0.16-0.39, *P* < .001), possibly because of prolonged abstinence for solid food, particularly indigestible solids, and the liquid diet that is prescribed for the day prior to the procedure (51, 101). Maselli et al (110), in a retrospective study of 57 adults on GLP-1RA-based therapy, reported that having a 24-hour liquid-only diet and a 12-hour fast resulted in no instances of retained gastric solids, pulmonary aspiration, gastro-oesophageal regurgitation, or hypoxia. The presence of microvascular or macrovascular complications of T2D was associated with a greater incidence of retained gastric contents (15% vs 2%, *P* < .01), as was use of insulin (17% vs 5%, *P* < .01) (104).

### T2D and Retained Gastric Content in the Absence of GLP-1 RA Treatment

Due to consequences of hyperglycemia (acute) and gastrointestinal autonomic neuropathy, including gastroparesis, in long-standing diabetes mellitus, the risk for retained gastric content associated with T2D (in the absence of treatment with GLP-1 RAs) needs to be considered. Baldawi et al published a meta-analysis of studies assessing residual gastric content by ultrasonography (111). They describe an approximately 2-fold higher prevalence of a “high-risk stomach” (increased antral cross-sectional area or gastric residual volume) in subjects with diabetes mellitus. Unfortunately, neither the type of diabetes mellitus nor the glucose-lowering medications used is described. It seems reasonable to assume that the majority of patients had T2D and that GLP-1 RAs were used in only a small fraction. Therefore, T2D, especially with long duration and accompanied by complications, may increase the risk for retained gastric content. Thus, not all the risk may has to be attributed to treatment with GLP-1RAs or tirzepatide.

### Endoscopy and the Risk of Pulmonary Aspiration

There have been multiple case reports of clinically significant aspiration following endoscopic procedures in people treated with GLP-1RAs, but findings on the magnitude of the risk have been conflicting. In the largest study by Yeo et al (112) of 963 184 individuals who underwent endoscopy, 46 935 (4.9%) were on GLP-1RA-based therapy and had a greater risk of aspiration pneumonia (HR 2.28, 95% CI 1.94-2.69, *P* < .0001) compared to those not on GLP-1RA-based therapy. This increased risk was evident after propensity-matched scoring of 59 factors that could affect gut motility or aspiration risk, with HR 1.33 (95% CI 1.02-1.74, *P* = .036). In another study by Barlowe et al (113), 6 806 045 unique upper endoscopy procedures were analyzed including 15 119 in GLP-1RA users, 14 407 in DPP-4 inhibitor users, and 7257 in chronic opioid users. There was no difference in the aspiration pneumonia risk with GLP-1RA use compared with either DPP-4 inhibitor use (relative risk 0.95, 95% CI 0.25-1.75) or chronic opioid use (relative risk 0.42, 95% CI 0.15-1.16); however, the incidence of aspiration pneumonia was rare, with 7 (0.05%), 10 (0.07%), and 8 (0.11%) cases in the GLP-1RA, DPP-4 inhibitor, and opioid arms, respectively. While chronic opioid use would be expected to slow gastric emptying, this would not be expected with the use of DPP-4 inhibitors. Figure 4B shows the results of studies reporting the risk of pulmonary aspiration in association with retained gastric contents. It can be estimated that of those with retained gastric contents, 0.2% had evident pulmonary aspiration.

### General Anesthesia and the Risk of Pulmonary Aspiration

Klonoff et al (114) evaluated the risk of postoperative complications in 13 661 people with diabetes, of whom 2256 (16.5%) were treated with a GLP-1RA (long-acting in 90% of cases) using high-dimensionality propensity score matching and found no increased risk of pulmonary aspiration in GLP-1RA users (0.54%) vs non-GLP-1RA users (0.69%), OR 0.78 (95% CI 0.29-2.09). Regarding the aspiration risk with emergency surgery, Dixit et al (115) studied 23 679 patients of whom 3502 (14.8%) had a prescription of GLP-1RA filled, although adherence to GLP-1RA treatment could not be confirmed in this study. The incidence of postoperative respiratory complications in GLP-1RA users (3.5%) and non-GLP-1RA users (4.0%) were comparable (OR 0.85, 95% CI 0.70-1.04, *P* = .12). However, this study is limited due to its small study size and poor specificity of the outcome measure.

In summary, in the largest study to date (112), GLP-1RA use was associated with a greater risk of aspiration pneumonia following endoscopy, but this finding was not replicated in other studies. With regard to such observational studies, several forms of bias may apply, which overall may impact the validity and reliability of findings and could lead to misinterpretations: Sampling bias occurs when the sample, whose results are measured, is not representative for the population it stands for; measurement bias refers to the inaccurate (eg, not appropriately standardized) data collection; observer/researcher bias describes the tendency to see what one expects to see (eg, in studies where the examiner was not blinded to the patients’ health status or treatment); publication bias describes the fact that results not compatible with the original hypothesis have a lesser chance to be submitted or accepted for publication (116). Potential confounders (T2D, obesity, associated conditions, and other medications) need to be taken into account. All studies on retained gastric content and pulmonary aspiration related to upper gastrointestinal endoscopy and induction of general anesthesia in subjects treated with GLP-1 RAs/incretin mimetics to date have been retrospective. Hence, prospective studies are required to quantify potentially increased risks under these circumstances.

### GLP-1 RA Treatment in Subjects Scheduled for Upper Gastrointestinal Endoscopy or General Anesthesia. Recommendations for Clinical Practice

There has been no consensus on the optimal management of individuals on GLP-1RA therapy undergoing either endoscopy or general anesthesia. The American Society of Anesthesiologists has suggested withholding short-acting GLP-1RAs for 1 day and long-acting GLP-1RAs for 1 week prior to the procedure (117). However, this may not be sufficient to reduce the risk of retained gastric contents and/or aspiration events, as the dose-response relationship suggests that even physiological concentrations of intact GLP-1 are sufficient to slow gastric emptying (16, 17). For example, steady-state plasma semaglutide concentrations with 1 mg per week injected subcutaneously have been reported to be approximately 40 nmol/L. Assuming that 99% is albumin-bound, only 1% (400 pmol/L) is free to diffuse into tissues and bind to GLP-1 receptors, but these concentrations are > 40-fold higher than physiological intact GLP-1 concentrations (118). With an elimination half-life of 7.6 days (119), pausing semaglutide injections for 1 week is expected to reduce plasma concentrations to approximately half this value, still >20-fold higher than physiological concentrations, which is sufficient to decelerate gastric emptying, as indicated by studies employing the GLP-1 receptor antagonist exendin (9-39) (17). To avoid retained gastric content, much longer withholding of semaglutide and other long-acting GLP-1RAs most likely would be needed, which would be impractical and may cause impaired perioperative glycemic control. In line with these considerations, the American Gastroenterology Association (120) did not find adequate evidence to support the cessation of GLP-1RA therapy in all patients and suggests that each patient requires an individual assessment to determine whether GLP-1RA therapy should be withheld. There was also the suggestion that in lieu of stopping GLP-1RAs, patients could be placed on a liquid diet 1 day prior to their procedure. An alternative suggestion was that for patients with symptoms suggesting retained gastric contents, for whom delaying endoscopy may have negative clinical consequences, rapid-sequence intubation should be considered. It is, however, unclear whether patients at risk can be identified through symptoms, as discussed earlier.

There may be a role for point-of-care gastric ultrasound as a noninvasive, cost-effective strategy to identify patients on GLP-1RA-based therapy with retained gastric contents (121, 122). However, no prospective studies have yet evaluated the effectiveness of this strategy to reduce the risk of pulmonary aspiration. If retained gastric contents are identified, the management pathways include (1) postponing the procedure, (2) proceeding with the procedure with precautions taken to prevent aspiration (eg, endotracheal intubation), or (3) accelerating gastric emptying pharmacologically. Intravenous erythromycin (∼3 mg/kg) has been shown to reverse the delay in gastric emptying induced by exogenous GLP-1 (123), and the same dose has been shown to initiate interdigestive motility in healthy human subjects (124). However, the effectiveness of this strategy to accelerate gastric emptying in individuals on GLP-1RA-based therapy has yet to be evaluated.

**Figure 7.**
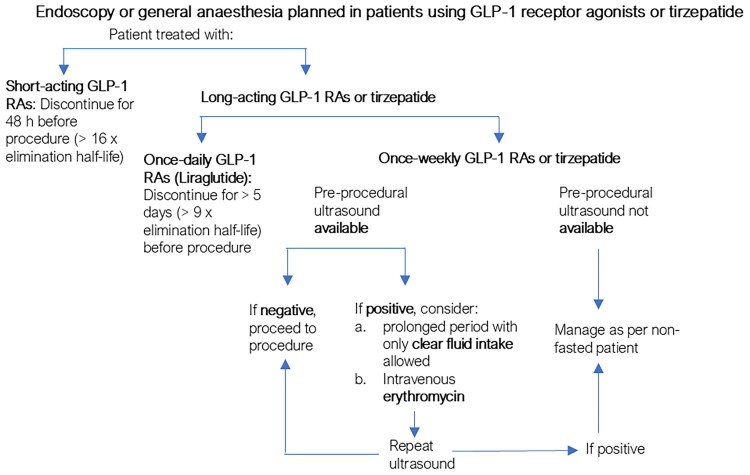
Clinical recommendations for subjects treated with short- or long-acting GLP-1RAs who are scheduled for upper gastrointestinal endoscopy or general anesthesia. The recommendations reflect current knowledge about the effects of GLP-1 RAs on gastric emptying and represent the opinion of the authors that has not been endorsed by any learned society. Abbreviations: GLP-1RA, glucagon-like peptide-1 receptor agonist.

Until more conclusive evidence becomes available, based on our understanding of the effect of GLP-1RA on physiology, we recommend the following (illustrated in Fig. 7):

1. There are insufficient data to support the cessation of GLP-1RAs preprocedurally. The American Society of Anesthesiologists’ suggestion to withhold short-acting GLP-1RAs for 24 hours and long-acting GLP-1RA for 1 week may not be enough to reduce the risk of aspiration. The optimal duration of cessation of GLP-1RAs to reduce aspiration risk is currently not known, but prolonged withholding of GLP-1RA-based therapy may have deleterious effects on glycemic control and weight management.
2. Where available, point-of-care gastric ultrasound could be considered to evaluate a patient's risk for aspiration. If retained gastric contents are identified on ultrasound prior to the procedure, it is reasonable to use IV erythromycin (∼3 mg/kg) and then reassess gastric content with ultrasound prior to commencing the procedure (123). If gastric ultrasound facilities and/or expertise is not available, patients on GLP-1RAs should be treated as unfasted and precautions taken to prevent pulmonary aspiration such as protecting the airway with endotracheal intubation.
3. A liquid diet for 1 day prior to the procedure may potentially reduce the risk of retained gastric contents, and therefore aspiration, but further studies in larger prospective trials are required before it can be generally recommended.

The following research questions can be identified for future studies to clarify unresolved issues: (1) What would be an appropriate duration of cessation of GLP-1RA therapy to avoid retained gastric content and aspiration? Individual answers may be required for various compounds in the class of incretin mimetic drugs. (2) How effective is pharmacological acceleration of gastric emptying (eg, with IV erythromycin) in individuals on GLP-1RA-based therapy and retained gastric content? (3) How effectively can a 24-hour period with intake only of clear fluids with 3 days of a low-residue diet reduce the risk of retained gastric contents and aspiration? Ideally, measurement of gastric emptying using an appropriate technique (eg, scintigraphy or stable isotope breath test) should be routine in clinical trials evaluating GLP-1RA-based therapy.

## Data Availability

Some or all datasets generated during and/or analyzed during the current study are not publicly available but are available from the corresponding author on reasonable request.
