# Correction to: “Clinical Consequences of Delayed Gastric Emptying With GLP-1 Receptor Agonists and Tirzepatide”

**DOI:** 10.1210/clinem/dgaf387

**Published:** 2025-07-10

In the above-named article by Jalleh RJ, Plummer MP, Marathe CS, Umapathysivam MM, Quast DR, Rayner CK, Jones KL, Wu T, Horowitz M, and Nauck MA (*J Clin Endo Metab*. 2025; 110(1): 1–15; doi: 10.1210/clinem/dgae719), there was an error in the References section.

In the originally published article, in the References section, reference 21 read as, “Jalleh RJ, Plummer MP, Marathe CS, et al. 2024. JCEM 2024 Online Supplement. figshare. https://doi.org/10.6084/m6089.figshare.26540737.v26540731. Journal contribution.” In the corrected article, the URL was updated to the reference and the reference reads as, “Jalleh RJ, Plummer MP, Marathe CS, *et al.* 2024. JCEM 2024 Online Supplement. *figshare*. https://doi.org/10.6084/m9.figshare.26540737.v1. Journal contribution.”

The article has been corrected online.

doi: 10.1210/clinem/dgae719

